# Exploiting Process-Related Advantages of Selective Laser Melting for the Production of High-Manganese Steel

**DOI:** 10.3390/ma10010056

**Published:** 2017-01-11

**Authors:** Christian Haase, Jan Bültmann, Jan Hof, Stephan Ziegler, Sebastian Bremen, Christian Hinke, Alexander Schwedt, Ulrich Prahl, Wolfgang Bleck

**Affiliations:** 1 Department of Ferrous Metallurgy, RWTH Aachen University, 52072 Aachen, Germany; jan.bueltmann@gmx.de (J.B.); jan.hof@iehk.rwth-aachen.de (J.H.); ulrich.prahl@iehk.rwth-aachen.de (U.P.); bleck@iehk.rwth-aachen.de (W.B.); 2 Fraunhofer-Institute for Laser Technology ILT, 52074 Aachen, Germany; stephan.ziegler@ilt.fraunhofer.de (S.Z.); sebastian.bremen@ilt.fraunhofer.de (S.B.); christian.hinke@ilt.fraunhofer.de (C.H.); 3 Central Facility for Electron Microscopy, RWTH Aachen University, 52074 Aachen, Germany; schwedt@gfe.rwth-aachen.de

**Keywords:** additive manufacturing, selective laser melting, steel, twinning, TWIP, martensite, TRIP, microstructure, texture, mechanical properties, anisotropy

## Abstract

Metal additive manufacturing has strongly gained scientific and industrial importance during the last decades due to the geometrical flexibility and increased reliability of parts, as well as reduced equipment costs. Within the field of metal additive manufacturing methods, selective laser melting (SLM) is an eligible technique for the production of fully dense bulk material with complex geometry. In the current study, we addressed the application of SLM for processing a high-manganese TRansformation-/TWinning-Induced Plasticity (TRIP/TWIP) steel. The solidification behavior was analyzed by careful characterization of the as-built microstructure and element distribution using optical and scanning electron microscopy (SEM). In addition, the deformation behavior was studied using uniaxial tensile testing and SEM. Comparison with conventionally produced TRIP/TWIP steel revealed that elemental segregation, which is normally very pronounced in high-manganese steels and requires energy-intensive post processing, is reduced due to the high cooling rates during SLM. Also, the very fast cooling promoted ε- and α’-martensite formation prior to deformation. The superior strength and pronounced anisotropy of the SLM-produced material was correlated with the microstructure based on the process-specific characteristics.

## 1. Introduction

During the last 30 years Additive Manufacturing (AM), also termed 3D printing, has increased significantly in importance in both industry and academia. AM techniques, such as Selective Laser Melting (SLM), Laser Metal Deposition (LMD), and Electron Beam Melting (EBM), which are currently the most widely used methods for AM of metals, have reached a level of technical maturity that allowed advancement from rapid prototyping of single pieces to final part production. Usually, AM parts are first defined in geometry by a computed 3D model and then built up layer upon layer, as opposed to subtractive or formative techniques. Recent developments in AM equipment, part quality, increased flexibility in terms of part geometry, enhanced demand for individualized production, reduced material waste, and energy usage are only a few advantages of AM that are responsible for the latest boom leading to a projected increase in material sales to $9 billion by 2026 [1,2,3,4].

Research in the field of AM of metals has mainly focused on materials and parts that are either difficult to manufacture, e.g., in terms of machining, or used in sectors with rather high-cost applications, e.g., for aerospace and biomedical applications. Therefore, particularly Al, Ti, Ni-base alloys, and tool steels have been investigated intensively [5,6,7,8]. However, advances in the direction of low-cost AM machines as well as scaled up productivity will undoubtedly result in the use of established, as well as adapted, metallic alloys in various markets [1,9]. High-Manganese Steels (HMnS) may be promising candidates to be used for AM [10,11]. This class of steels is characterized by outstanding mechanical properties, but wide industrial application, e.g., as material for automobile sheets, has been impeded so far due to their comparatively high alloying and processing costs. The general concept of HMnS is based on stabilizing the face-centered cubic austenite phase with a high amount of Mn (15–30 wt %) and additions of C (0.05–1 wt %) and Al (0–3 wt %) [12]. As a result, HMnS exhibit low stacking fault energy (SFE) values in the range between ~10 and ~50 mJ/m^2^ at room temperature [13,14]. The low SFE is accountable for activation of additional deformation mechanisms, such as deformation twinning and martensite formation that promote the TWinning-Induced Plasticity (TWIP) and TRansformation-Induced Plasticity (TRIP) effects. As a consequence, HMnS show very high strain hardenability that can be tailored within a wide range by adjusting the chemical composition and microstructure [15,16,17,18,19,20].

The aim of the current study was first to identify the applicability of AM for the production of fully dense and high-quality HMnS bulk samples. Second, the influence of process-inherent characteristics on the microstructure evolution and mechanical properties was investigated. Specifically, the SLM technique was used to build up bulk material from prealloyed X30Mn22 powder feedstock. The as-built material was characterized with respect to microstructure, element distribution, and mechanical properties. The solidification and deformation behavior of the SLM-produced steel was compared with conventionally processed counterparts. Based on these results, the process-microstructure-property relations were discussed.

## 2. Experimental Procedure

### 2.1. Material Chemistry and Processing

**Figure 1:**
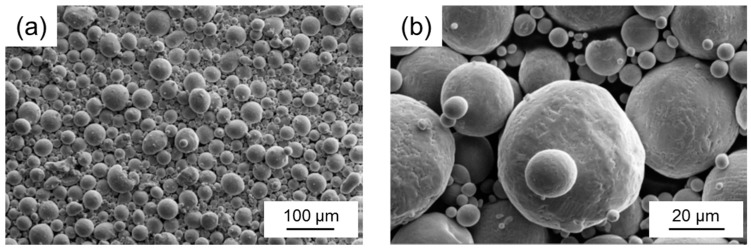
(**a**) Low and (**b**) high magnification SEM image of the prealloyed powder used for sample production by SLM.

The chemical composition of the TRIP/TWIP steel investigated in this study is given in Table 1. The material was first ingot-cast, followed by electrode induction melting gas atomization (EIGA) using argon as the atomizing medium to produce the prealloyed powder. The powder particles constituted a spherical shape and were sieved to guarantee a size below 45 µm (Figure 1). During the SLM process, which will be described in the following, the chemical composition was mainly altered due to vaporization of manganese (Table 1). The SFE of the material after SLM was calculated to be ~10 mJ/m^2^ using a subregular solution thermodynamic model [21].

**Figure 2:**
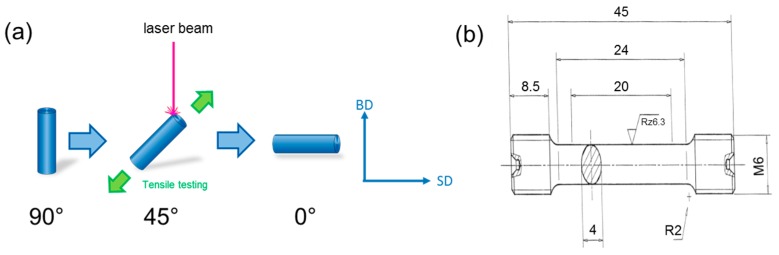
(**a**) Schematic illustration of the build-up process of the cylindrical billets for tensile specimens. The angles denote the position of the tensile direction with respect to the scan direction (SD). BD denotes the build-up direction; (**b**) Geometry (in mm) of the tensile samples used for mechanical testing (DIN 50125).

**Figure 3:**
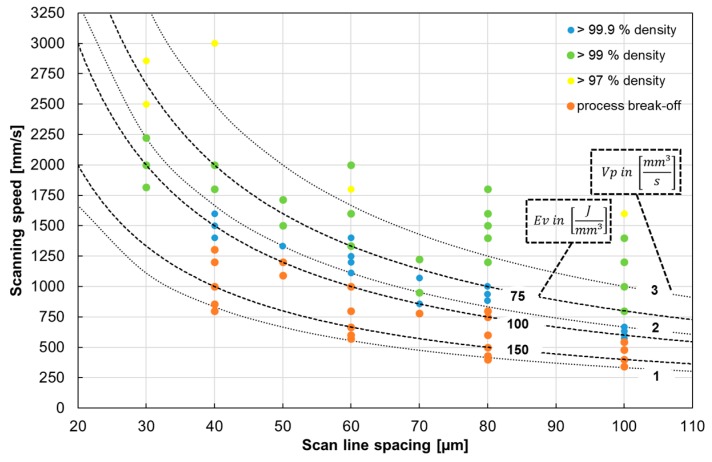
Material density after SLM, depending on scanning speed and scan line spacing. *V_p_* and *E_v_* denote build-up rate and energy density ($V_{p}=D_{S}\cdot v_{S}\cdot \Delta y_{S}$, $E_{v}=\frac{P_{L}}{D_{S}\cdot v_{S}\cdot \Delta y_{S}}$ . Here *D_S_* denotes the layer thickness, *v_S_* the scan speed, Δ*y_S_* the scan line spacing, and *P_L_* the laser power), respectively.

Samples for density and microstructure analysis (10 mm^3^ cubes), as well as for tensile testing (cylindrical rods with 45 mm length and 6 mm diameter), were built using a M1 cusing SLM device (Concept Laser GmbH) equipped with a Yb fiber laser. Thin-walled (0.5 mm) supporting structures were only employed at the bottom of each cube and rod for easier removal from the substrate plate. In order to investigate the anisotropy of the mechanical properties the billets for tensile specimens were built with the tensile direction being 90°, 45°, and 0° to the scan direction (SD) (see Figure 2). A constant laser power of 180 kW, focus diameter of 60 µm, and layer thickness of 30 µm were used. A scan strategy with bidirectional laser beam movement within each layer and a rotation of 90° between subsequent layers was chosen. Optimized process parameters for production of dense bulk material (*ρ* ≥ 99.9% as obtained by optical analysis of residual porosity) were identified by systematic variation of scan speed and scan line spacing (Figure 3). On the one hand, using high energy densities (>100 J/mm^3^) led to the formation of spillings on top of both the previously built-up material and the powder bed. Therefore, the process was stopped manually (process break-off). On the other hand, low energy densities and high build-up rates caused insufficient melting of the powder particles and returned material with density <99.9%. Based on this analysis a scan line spacing of 100 µm and scan speed of 571 mm/s were chosen to build up cylinders for the machining of tensile specimens.

### 2.2. Specimens and Characterization Techniques

Specimens for microstructure characterization were cut using electron discharge machining. Sample preparation consisted of mechanical grinding up to 1200 SiC grit paper, followed by polishing using 3 µm and 1 µm diamond suspension on a Struers Abrapol-2. For optical microscopy and scanning electron microscopy (SEM) analysis, the specimens were further etched at room temperature using a 2% Nital solution (95 mL C_2_H_5_OH and 5 mL HNO_3_). For electron backscatter diffraction (EBSD), the mechanically polished surface was also electropolished at room temperature for 20 s at 22 V. The used electrolyte consisted of 700 mL ethanol (C_2_H_5_OH), 100 mL butyl glycol (C_6_H_14_O_2_), and 78 mL perchloric acid (60%) (HClO_4_). All measurements were performed on the BD-TD planes (BD-build-up direction, TD-transverse direction (transverse to SD)).

Energy dispersive X-ray spectroscopy (EDX) and EBSD analyses were conducted using a JEOL JSM 7000F FEG-SEM with an EDAX-TSL Hikari detector and OIM DataCollection 7.3/OIM Analysis 7.3 software (EDAX Inc., Mahwah, NJ, USA). Measurements were performed at a voltage of 20 kV, with a probe current of approximately 30 nA and a step size of 200 nm. Evaluation of the EBSD data was performed by considering all scanned points with a confidence index (CI) between 0.1 and 1.0 [22]. The criterion for the definition of ∑3 grain boundaries was 60° misorientation about the <111> axis, with an angular tolerance of 5° within the austenitic (face-centered cubic) matrix.

The mechanical properties of the material were evaluated by uniaxial tensile testing at room temperature and a constant strain rate of 10^−3^·s^−1^ on a universal tensile testing machine of type Z4204 manufactured by Zwick/Roell equipped with a 50 kN load cell. According to DIN 50125, the round-shaped specimens used for uniaxial tensile tests had a 20 mm gauge length and 4 mm diameter (Figure 2b).

## 3. Results and Discussion

### 3.1. Solidification Behavior

**Figure 4:**
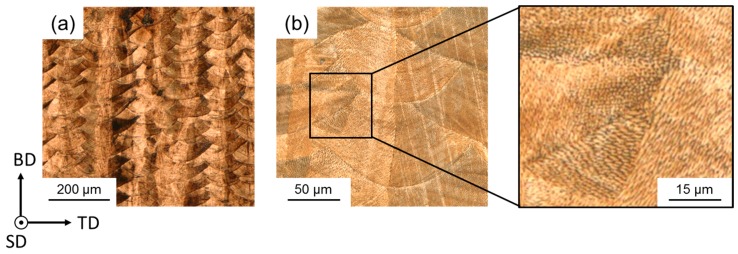
Optical micrographs of the investigated steel after SLM. (**a**,**b**) reveal the morphology of laser-melted tracks in the BD-TD plane. In addition, a fine cellular dendrite structure may be observed in the inset in (**b**).

**Figure 5:**
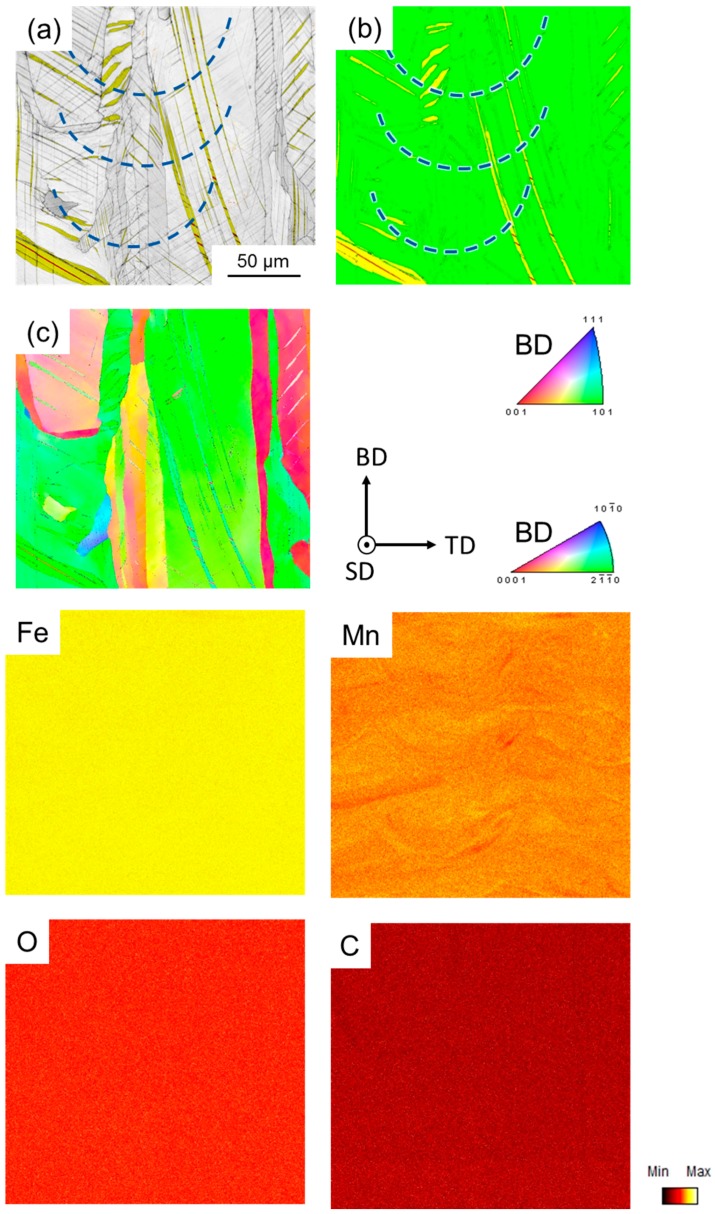
Microstructure of the investigated steel after SLM: (**a**) image quality EBSD map overlain by a phase map only including hexagonal ε-martensite in yellow and tetragonal α’-martensite in red; (**b**) EBSD map showing the autenite phase in green, hexagonal ε-martensite in yellow, and α’-martensite in red; (**c**) inverse pole figure EBSD map, and Fe, Mn, O, and C EDX element maps taken from the same area as (**a**–**c**). The blue dashed lines in (**a**,**b**) indicate the position of individual laser-melted tracks.

The microstructure of the investigated steel after processing by SLM is depicted in Figure 4 and Figure 5. Figure 4a illustrates the typical structure resulting from layer-wise buildup of bulk material by individual parallel (in TD) and stacked (in BD) laser-melted tracks. In addition, elongated features, mainly occurring in BD, that were not restricted in their extension by the edges of the laser-melted tracks were present (Figure 4b). Furthermore, a fine cellular dendrite structure of varying morphology and growth direction, due to varying local solidification conditions, may be observed (inset in Figure 4b). From Figure 5a and c it became evident that the elongated features revealed the underlying grain structure, with most grains elongated parallel to BD. This is in agreement with the preferred solidification in the direction of heat flow [23] and with the work by Thijs et al. [24], who also reported a fine dendritic structure with an underlying directionally solidified grain structure in a highly-alloyed, SLM-processed, face-centered cubic Al alloy. The fine dendritic structure in the investigated steel indicates short segregation lengths, i.e., absence of macro segregations, and, thus, homogeneous element distribution, which facilitates small variation of the local SFE. Although the local conditions of heat transfer within the melt pool are quite complex and depend on several factors such as melt pool size, heat conductivity of the surrounding bulk metal, powder, and atmosphere, the main proportion of the induced heat is usually directed towards the substrate plate [25,26]. As a consequence, the majority of grains in the present microstructure were characterized by a high aspect ratio with largest dimension in BD, where epitaxial growth further promoted the extension of grains over several laser-melted layers. With respect to the crystallographic microtexture, the grains present in the area of the EBSD map were dominantly oriented close to <001> and <101> with respect to BD (Figure 5c), which is consistent with the findings in [24]. However, it must be noted that the EBSD map contained only a few grains, and, therefore, the macrotexture may be deviating.

In addition to the rather typical microstructure and texture, the investigated steel surprisingly was found to contain not only the face-centered cubic austenite phase but also small portions of the tetragonal α’-martensite located in hexagonal ε-martensite laths (Figure 5a,b). Under conventional cooling conditions, e.g., during ingot casting, ε- and α’-martensite, as thermodynamically metastable phases, are usually not formed during the solidification and subsequent cooling of high-manganese steel, although the austenite phase is also metastable at room temperature. However, according to [27] ε-martensite can, on the one hand, be formed locally even in the as-cast state in areas of low Mn and C content, and thus low SFE, due to pronounced segregation. On the other hand, ε-martensite was also found to be induced under conditions of very high cooling rates during laser spot welding [27]. In the present steel, the ε-martensite laths extended over several laser-melted layers (Figure 5a,b), i.e., they were not restricted in size by these layers but only by the grain boundaries of the austenite grains, and the alloying elements were distributed homogeneously except for slight Mn segregation (Figure 5, element mappings). Regarding the cooling conditions, extremely high cooling rates at the order of 10^6^ K/s may be realized during SLM [28]. Consequentially, high internal stresses and dislocation densities are present in additively manufactured metals [29,30]. It can therefore be concluded that the austenite-to-ε-martensite transformation contributed to partial compensation of the internal stresses and was induced due to the high cooling rate, whereas α’-martensite was only formed within the hexagonal martensite phase following the γ => ε => α’ transformation reaction [31]. Also, the relatively large grain size, compared to recrystallized samples, and the decreased austenite stability due to loss of Mn during SLM (Table 1) further facilitated ε-martensite formation [32,33,34].

**Figure 6:**
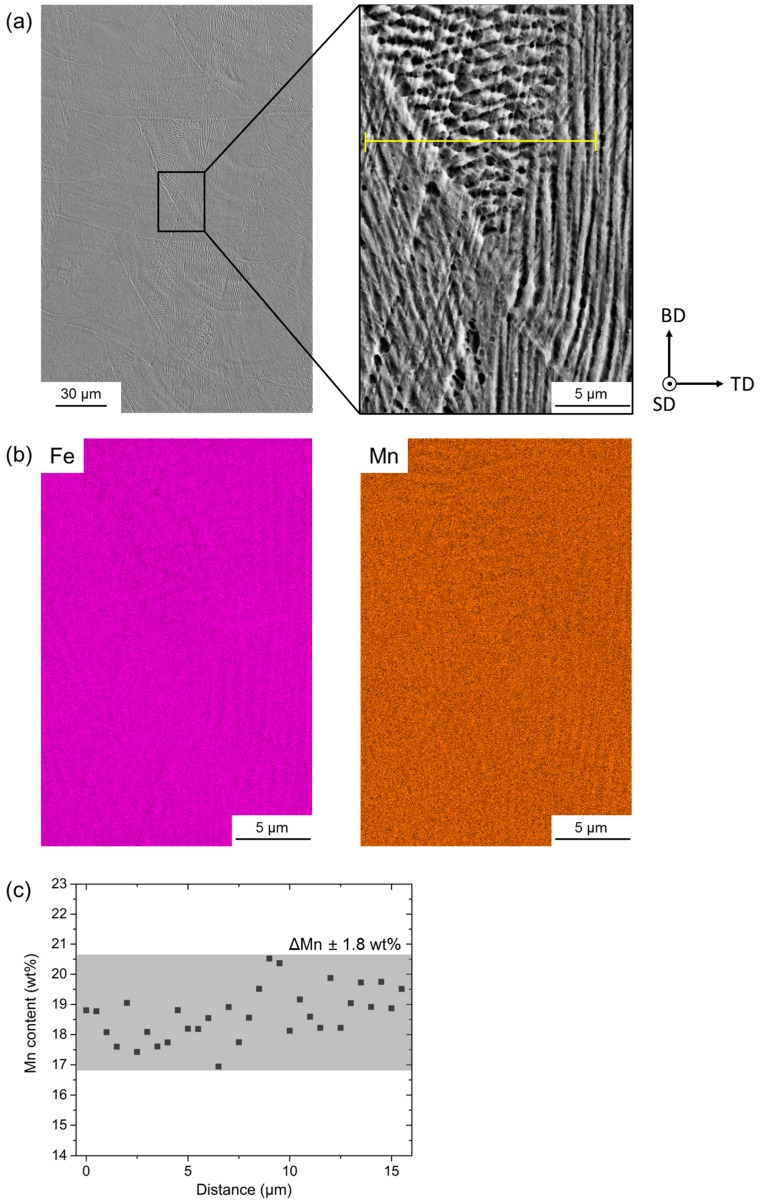
(**a**) SE image of the investigated steel after SLM. The enlarged area in (**a**) contains the edge of a laser-melted track (top left to bottom right corner of inset). (**b**) Fe and Mn EDX element maps taken from the same area as the inset in (**a**). The yellow line in the inset in (**a**) marks the position of an EDX line scan. The corresponding Mn distribution is shown in (**c**).

**Figure 7:**
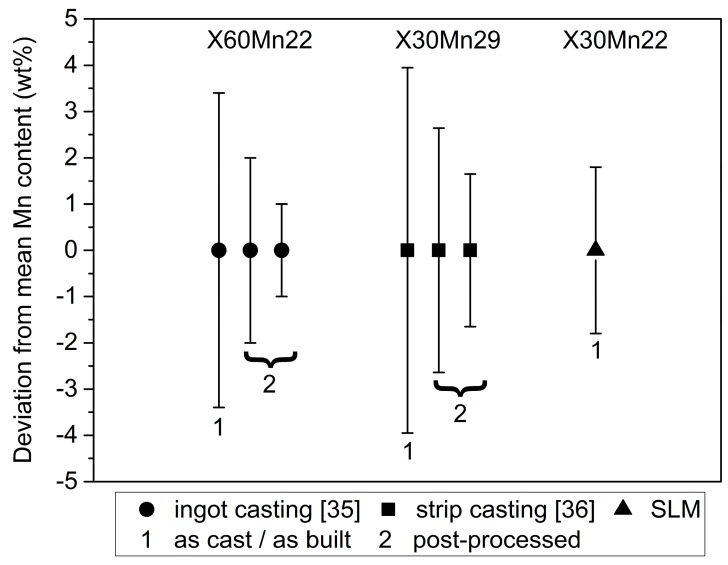
Comparison of the deviation of the Mn content from the mean Mn value depending on the processing route. An X60Mn22 (ingot casting) [35] and an X30Mn29 (strip casting) [36] steel were used for comparison. The Mn deviation after both ingot and strip casting is much higher compared to the as-built SLM material. The ingot-cast steel was post-processed by forging and annealing (middle circle) as well as additional hot rolling (right circle). The strip-cast steel was post processed by cold rolling and annealing at 900 °C for 30 min (middle square) and at 1150 °C for 30 min (right square).

As mentioned above, the EDX element mappings in Figure 5 revealed a homogeneous distribution of the contained alloying elements; no detectable formation of oxides, which would be detrimental to the mechnical properties; and only slight segregation of Mn at edges of laser-melted tracks. Figure 6a shows such an area containing several laser-melted layers. The inset in Figure 6a shows again the fine dendritic structure, as also illustrated in Figure 4b. The respective Fe and Mn EDX element maps in Figure 6b revealed that the elements were distributed homogeneously with slight enrichment of Fe and depletion of Mn within the dendrites, and vice versa between the dendrites. Analysis of the Mn content along the line in the inset of Figure 6a revealed that the Mn content varied by ±1.8 wt % (Figure 6c). Although the Mn variation in the SLM-produced steel was measured over a short distance and needs further verification, this value was lower compared to the Mn variation in as-cast HMnS produced by ingot [35] and strip casting [36,37], as shown in Figure 7. Only the application of energy-intensive post processing allows the reduction of element segregation and the achievement of the Mn distribution detected in the SLM-processed steel. Hence, HMnS, which are usually prone to strong segregation of alloying elements, are promising candidate materials to utilize the process-specific advantages of SLM and other AM techniques.

### 3.2. Deformation Behavior

**Figure 8:**
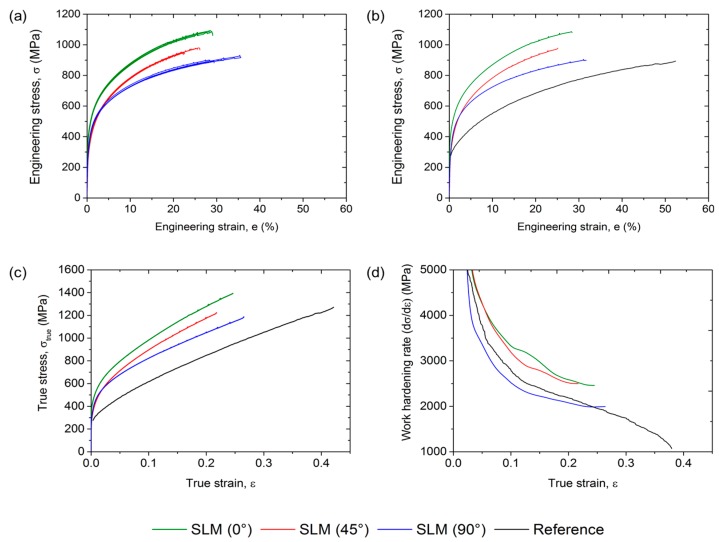
(**a**) Engineering stress-strain curves of all SLM-produced samples tested; (**b**) engineering stress-strain curves of selected SLM-produced samples of each condition in comparison with a reference steel; (**c**) true stress-true strain curves; and (**d**) work hardening rate-true strain curves. The reference sample refers to an X30Mn22 steel processed conventionally, whereas 90°, 45°, and 0° refer to the angle between tensile direction and scan direction, as shown in Figure 2.

**Figure 9:**
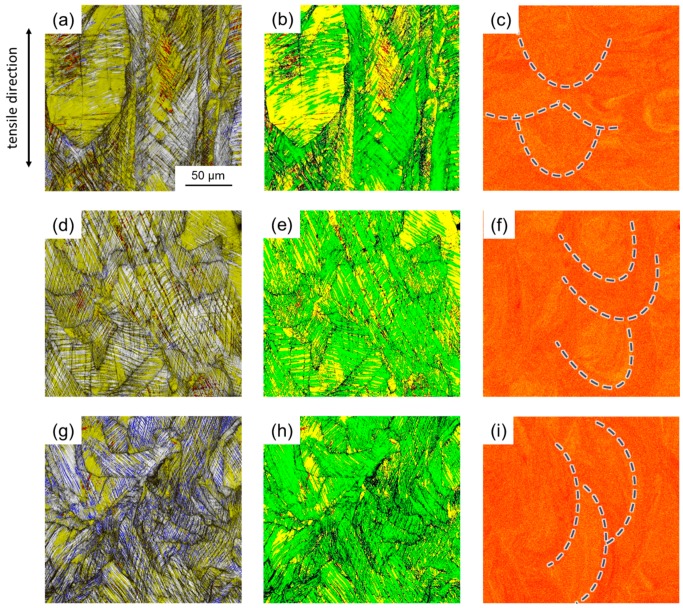
Microstructure of the specimens after tensile loading (up to fracture): (**a**–**c**) 90°; (**d**–**f**) 45°; (**g**–**i**) 0°. (**a**,**d**,**g**) are image quality EBSD maps overlain by a phase map only including hexagonal ε-martensite in yellow and tetragonal α’-martensite in red. Blue lines indicate Σ3 grain boundaries (60° ± 5° <111>). (**b**,**e**,**h**) are EBSD maps showing the autenite phase in green, hexagonal ε-martensite in yellow, and α’-martensite in red. (**c**,**f**,**i**) illustrate EDX element maps of the Mn distribution. Blue dashed lines indicate the position of individual laser-melted tracks.

**Figure 10:**
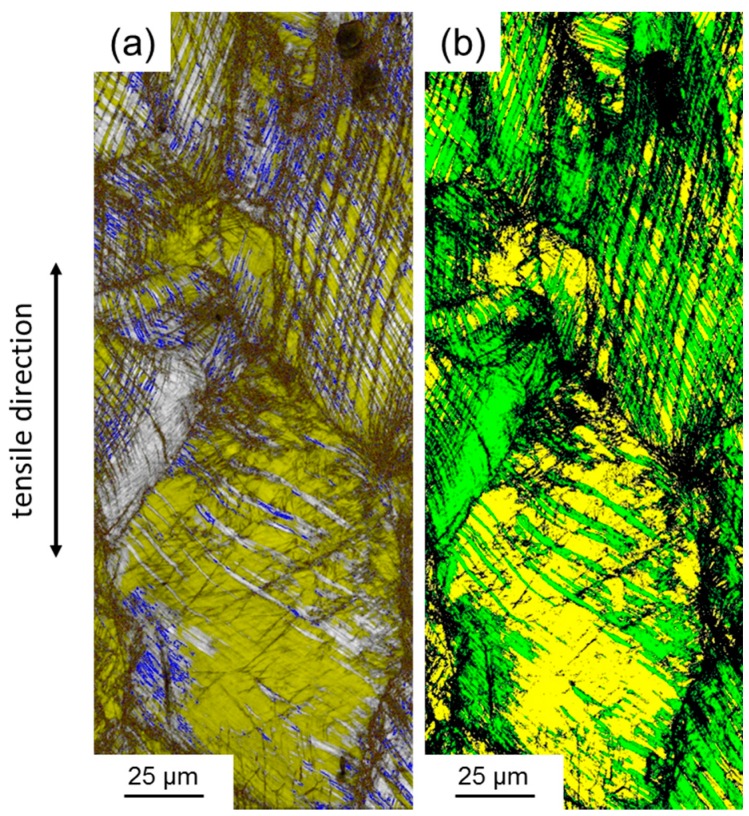
(**a**) Image quality EBSD map overlain by a phase map only including ε-martensite, α’-martensite, and Σ3 grain boundaries and (**b**) EBSD phase map of a X30Mn22 after conventional processing and tensile deformation until fracture. The color-coding is the same as in Figure 6. (In contrast to the measurements on the SLM samples, this measurement has been performed using a DigiView III camera, OIM V. 5.2, and 25 keV electron energy—the comparability to the newer measurements at this scale, however, was not affected by the different settings).

The mechanical properties of the investigated steel are shown in Figure 8 and summarized in Table 2 in comparison with a conventionally processed steel of the same chemical composition. First of all, all tensile samples produced using SLM are included in Figure 8a. It is obvious that the five samples of each condition, i.e., 0°, 45°, and 90° angle between scan direction and tensile axis, showed very similar deformation behavior, which is also indicated by the low scatter of the mechanical properties (Table 2). Therefore, the processing conditions and parameters established in this study allowed for the production of HMnS with reproducible and reliable properties. Comparison between the SLM-produced material and the reference material revealed that the strength of the additively manufactured samples was higher and the elongation lower than the corresponding values of the conventionally produced steel (Figure 8b,c). The high initial dislocation density as well as the presence of ε- and α’-martensite after SLM facilitated the higher strength, whereas residual porosity and impurities, even if present to a very low extent, had a detrimental effect on the formability, as often observed in powder metallurgically produced metals. However, the slope of the true stress-strain curves and of the work hardening rate-true strain curves showed that the same deformation characteristics, i.e., high work hardening capacity and the linear increase in true stress, were obtained regardless of the processing route. This is further evidenced by the presence of Σ3 grain boundaries (deformation twins), ε-, and α’-martensite, after tensile testing as a result of activation of the TRIP and TWIP effects (Figure 9 and Figure 10). It is also important to note that the small fractions of ε- and α’-martensite present after the SLM process did not result in a decreased work-hardening rate, as compared to the conventionally produced materials, i.e., the full potential of the material was available during deformation of the SLM-produced samples.

From Figure 8a strongly anisotropic behavior of the SLM-produced steel was obtained. With an increasing angle between scan direction and tensile direction the work hardening rate and the strength of the material decreased. This is in contrast to conventionally produced HMnS after recrystallization annealing due to their equiaxed grain structure and weak crystallographic texture [36,38,39]. In principle, two features can be accountable for the anisotropy of the SLM-produced material: (i) the relative position of the laser-melted tracks with respect to the tensile direction; and (ii) the underlying microstructure. With respect to (i), it was described above that material with a density >99.9% was used exclusively and that only slight segregation of manganese at the edges of the individual tracks was observed. Therefore, anisotropic distribution of pores and strong segregation of manganese were not responsible for the anisotropy. In addition, both grain growth during solidification (Figure 5) as well as deformation-induced formation of twins, ε-, and α’-martensite during tensile testing (Figure 9) were neither restricted nor suppressed by the orientation and size of the laser-melted tracks. Thus, it can be concluded that the relative position of the individual tracks was not responsible for the anisotropy of the mechanical properties. With respect to (ii), the microstructure formed during SLM was found to be highly anisotropic in its nature. Although the crystallographic texture is usually weaker if a rotation in subsequent layers is applied [24], and despite the fact that the preferred orientation in the investigated steel was much less compared to other studies [40,41], the texture formed in the present material can be expected to be more pronounced compared to conventionally processed HMnS, which are typically characterized by a weak texture [39,42]. In addition, also a strong morphological texture containing grains preferentially elongated parallel to BD was observed. On the one hand, the effective grain size in tensile direction in the 90° samples was much larger compared to the 0° samples. This clearly supported the higher yield strength of the latter. On the other hand, following the model of Byun [43], the equivalent critical uniaxial stress for activation of deformation twinning can be calculated by

(1) $$\sigma_{twin}=6.14\frac{\gamma_{SF}}{b_{\mathrm{SP}}},$$

where γ_SF_ = 10 mJ/m^2^ and *b*_SP_ = 14.5 nm [44] are the SFE and Burgers vector of the Shockley partial dislocations, respectively. The calculated critical stress in this case is 423 MPa. This value is only slightly above the yield strength of the 0° sample. Hence, the high initial strength required for plastic flow of the 0° material would lead to the activation of deformation twinning at lower plastic strains, which, in turn, results in a higher contribution of twinning to the accomodation of plastic strain, as compared to the 90° sample. As a consequence, the strength and work hardening rate of the 0° material were presumably enhanced due to this increased contribution of deformation twinning. Furthermore, the determined Σ3 grain boundary fraction, which decreased with an increasing angle between scan direction and tensile direction, also promoted the superior work hardening of the 45° sample compared to the 90° condition. However, it must also be noted that the EBSD data was aquired from a small area and, thus, was statistically not reliable. Furthermore, σ_twin_ was calculated using an average Schmid factor of 0.326 as a first approximation. A pronounced texture would certainly influence the critical stress for dislocation movement, martensite, and twin formation. Here, a more thorough analysis of the correlation between crystallographic texture and activation of the different deformation mechanisms, which may also include consideration of anisotropic residual stresses and variant selection based on mechanical work criteria, is needed to fully understand the deformation behavior of the SLM-produced HMnS. These factors will be addressed in a separate study.

Although the details of the correlation between microstrcuture, texture, and deformation behavior need further detailed analyses, it can be stated that the superior strength of SLM-produced HMnS, in addition to the dependence of activated deformation mechanisms on the morphological and crystallographic texture, makes these materials promising candidates for AM parts. Local control of the grain structure and texture, which can be achieved by varying processing parameters, enables differing contributions of slip, TRIP, and TWIP and, thus, allows tailoring of the local work hardening. These effects can be enhanced further by using chemical gradients and are currently under investigation by the authors. For instance, density-reduced parts with 3-dimensional lattice structures that require high strength and energy-absorption potential may be suitable applications for additively manufactured HMnS.

## 4. Conclusions

A high-manganese TRIP/TWIP steel was successfully produced using SLM of prealloyed powder. The solidification as well as the deformation behavior were investigated experimentally and compared with conventionally produced HMnS. The following conclusions can be drawn.

- The high cooling rates that are associated with the SLM process facilitated the production of HMnS with reduced elemental segregation compared to ingot- and strip-cast material. Therefore, energy-intensive post processing may be avoided and makes HMnS a promising candidate material to ideally use process-specific peculiarities.
- Whereas the orientation of laser-melted tracks was found to have no influence on the material properties, the morphological and crystallographic texture due to directional solidification affected the mechanical behavior significantly. Especially the anisotropy of the grain size and the varying contribution of deformation twinning depending on the angle between scan direction and tensile direction defined the work hardening of the steel.
- The SLM-produced samples obtained superior strength but reduced formability with high reproducibility. As well as in conventionally produced HMnS, TRIP and TWIP effects were activated in the additively manufactured HMnS and allowed for the beneficial work hardening of this class of steels.
- The as-built state already contained ε- and α’-martensite, induced as a result of the very fast cooling during SLM. The initial martensite fraction and dislocation density can be varied depending on the cooling rate and, thus, may be used to tailor the yield strength.

## Figures and Tables

**Table 1:** Chemical composition of the investigated steel after ingot casting and after sample production via SLM. All contents are given in wt %.

State	Fe	Mn	C	Al	Si	S	P
Ingot-cast	bal.	21.67	0.293	0.003	0.05	0.0085	0.01
After SLM	bal.	20.15	0.274	0.014	0.05	0.0105	0.01

**Table 2:** Mechanical properties of the investigated steel. YS, UTS, and e_pl_ denote yield strength, ultimate tensile strength, and total plastic elongation, respectively.

State	YS (MPa)	UTS (MPa)	e_pl_ (%)
SLM (0°)	416 ± 9	1085 ± 6	27.4 ± 1.2
SLM (45°)	302 ± 16	966 ± 14	23.8 ± 1.6
SLM (90°)	329 ± 3	906 ± 11	31.2 ± 2.9
Reference	275	894	52
